# Supplementary material for: Bioprotection Efficiency of Metschnikowia Strains in Synthetic Must: Comparative Study and Metabolomic Investigation of the Mechanisms Involved
Source: Foods. 2023 Oct 26;12(21):3927. doi: 10.3390/foods12213927 (PMC10649255; doi:10.3390/foods12213927)
Supplement: Supplementary file 1 [file foods-12-03927-s001.zip › foods-2647085-supplementary.pdf]

Table S1. Eluant gradient for amino acids analyses by HPLC

Time (min)	Mobile phase A (%)	Mobile phase B (%)
**0.00**	98	2
**0.25**	98	2
**9.40**	47	53
**9.50**	0	100
**16.50**	0	100
**16.51**	98	2
**18.50**	98	2

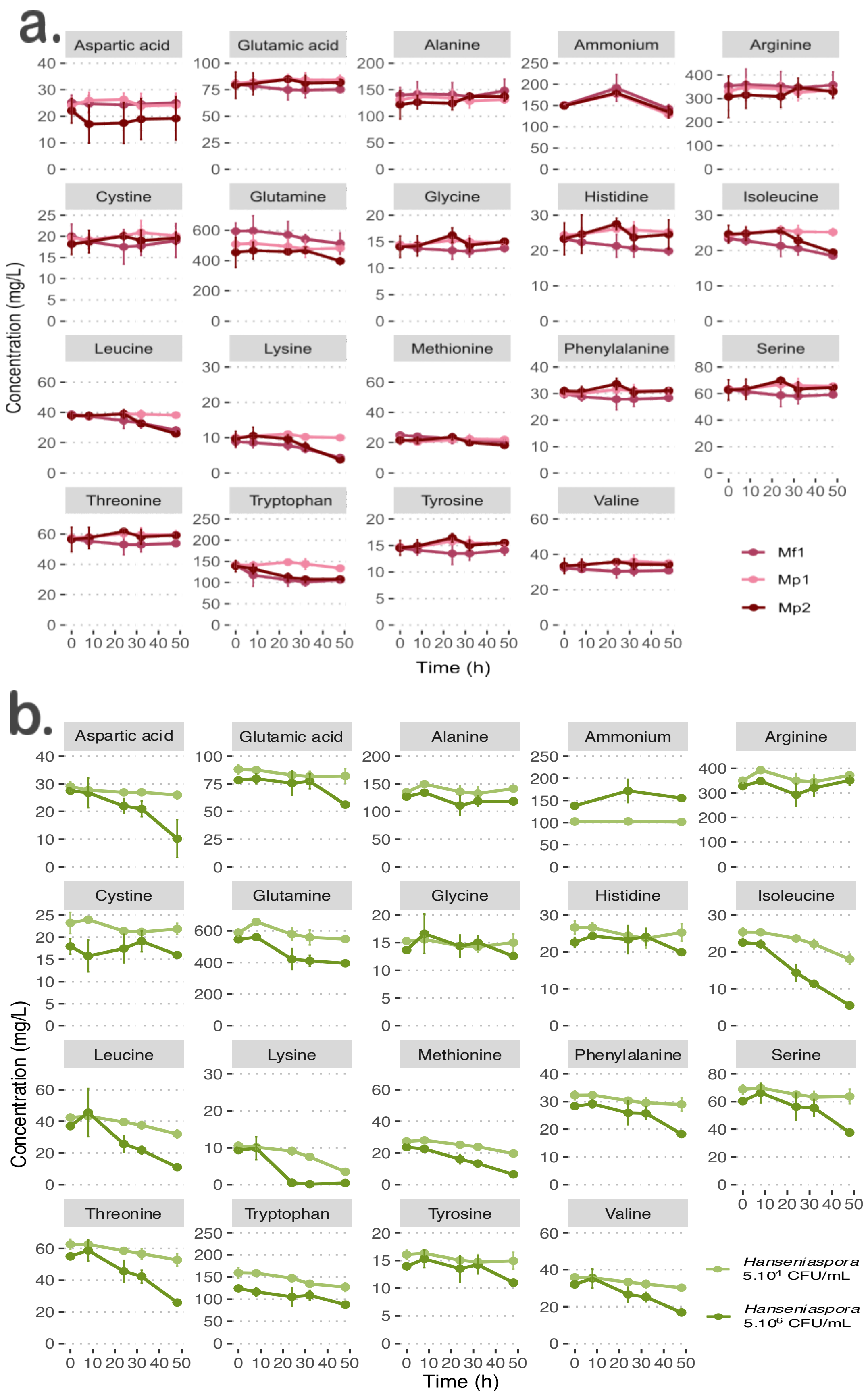

Figure S1. Nitrogen consumption at 12 °C in single culture (A) of the two *M. pulcherrima* strains (Mp1 and Mp2) and the *M. fructicola* strain (Mf1), and (B) of *Hanseniaspora* at an initial concentration of 5.10^{4} CFU/mL (light green) and at 5.10^{6} CFU/mL (dark green).

Table S2. Concentration in ammonia (mg/L) and total amino acids ($mgNH_2/L$) in co-culture with Mp2 and Mf1.

		Ammonia (initial concentration 155mg/L)	Amino acids (initial concentration 206 $mgNH_2/L$)
Hanseniaspora at 5.10^4 CFU/mL	with Mp2	164,53 ± 49,60	147,50 ± 1,87
	with Mf1	185,73 ± 51,89	142,20 ± 2,55
Hanseniaspora at 5.10^6 CFU/mL	with Mp2	127,03 ± 4,91	173,00 ± 40,73
	with Mf1	161,53 ± 47,07	179,70 ± 43,68

Mp2

	% 8h	% 24h	% 48h	Lag	Qs	Qs48
Ile	0.52	2.18	20.38	21.00	4,70E-02	4,57E-03
Leu	0.29	3.04	31.20	20.00	9,30E-02	1,05E-02
Lys	1.14	4.99	60.40	10.00	6,70E-02	5,27E-03
Met	0.46	0.90	13.97	24.33	6,70E-02	2,83E-03
Trp	4.86	17.91	21.61	7.33	4,50E-01	2,73E-02

Mf1

	% 8h	% 24h	% 48h	Lag	Qs	Qs48
Ile	2.62	8.86	20.92	11.67	6,80E-02	8,99E-03
Leu	3.41	10.91	27.06	7.67	1,30E-01	1,94E-02
Lys	2.73	11.27	50.97	7.00	3,10E-02	8,09E-03
Met	3.30	9.34	17.48	9.67	8,00E-02	8,18E-03
Trp	16.79	24.95	24.72	2.67	2,30E+00	6,38E-02

***Hanseniaspora* 5.10^4 CFU/mL**

	% 8h	% 24h	% 48h	Lag	Qs	Qs48
Ile	2.40	7.00	28.67	15.00	1,90E+00	7,52E-03
Leu	2.09	6.98	24.26	15.67	4,60E+00	1,08E-02
Lys	5.89	12.44	66.18	15.00	1,40E+00	7,15E-03
Met	0.40	8.04	27.79	17.67	1,70E+00	7,75E-03
Trp	2.91	8.15	19.49	14.67	1,50E+01	3,34E-02

***Hanseniaspora* 5.10^6 CFU/mL**

	% 8h	% 24h	% 48h	Lag	Qs	Qs48
Ile	2.11	36.62	75.54	7.33	2,30E-02	2,07E-03
Leu	0.41	30.96	70.13	6.67	2,10E-01	3,15E-03
Lys	8.01	94.72	95.24	6.00	4,40E-02	1,08E-03
Met	4.52	32.19	72.65	7.33	3,60E-02	2,09E-03
Trp	6.81	15.78	29.38	11.67	2,30E-01	4,49E-03

Figure S2. Table with consumption parameters: % 8h, % 24h and % 48h correspond to percentage of consumption after 8, 24 and 48 h of growth, respectively, Lag corresponds to the time of the lag phase before the consumption of nitrogen (h), Qs corresponds to maximal consumption speed (ng/L/h/cells), Qs48 corresponds to global consumption speed between 0 and 48 h of growth (ng/L/h/cells).

Table S3 | Oxygen consumption in synthetic must (MS300) at 12 °C

Strain	Initial concentration (CFU/mL)	Time (h)[1]	Speed (mg/L/h)[2]
Mp1	1.10^6	1.68 ±0.00[a]	0.091 ±0.003[a]
Mp2	1.10^6	2.45 ±0.38[ab]	0.058 ±0.007[ab]
Mf1	1.10^6	3.06 ±0.19[ab]	0.048 ±0.003[ab]
Hans	1.10^4	34.33 ±0.00[b]	0.014 ±0.000[b]
Hans	1.10^6	3.28 ±0.19[ab]	0.05 ±0.005[ab]

[1]Time needed to deplete the medium of oxygen
[2]Maximal oxygen consumption speed

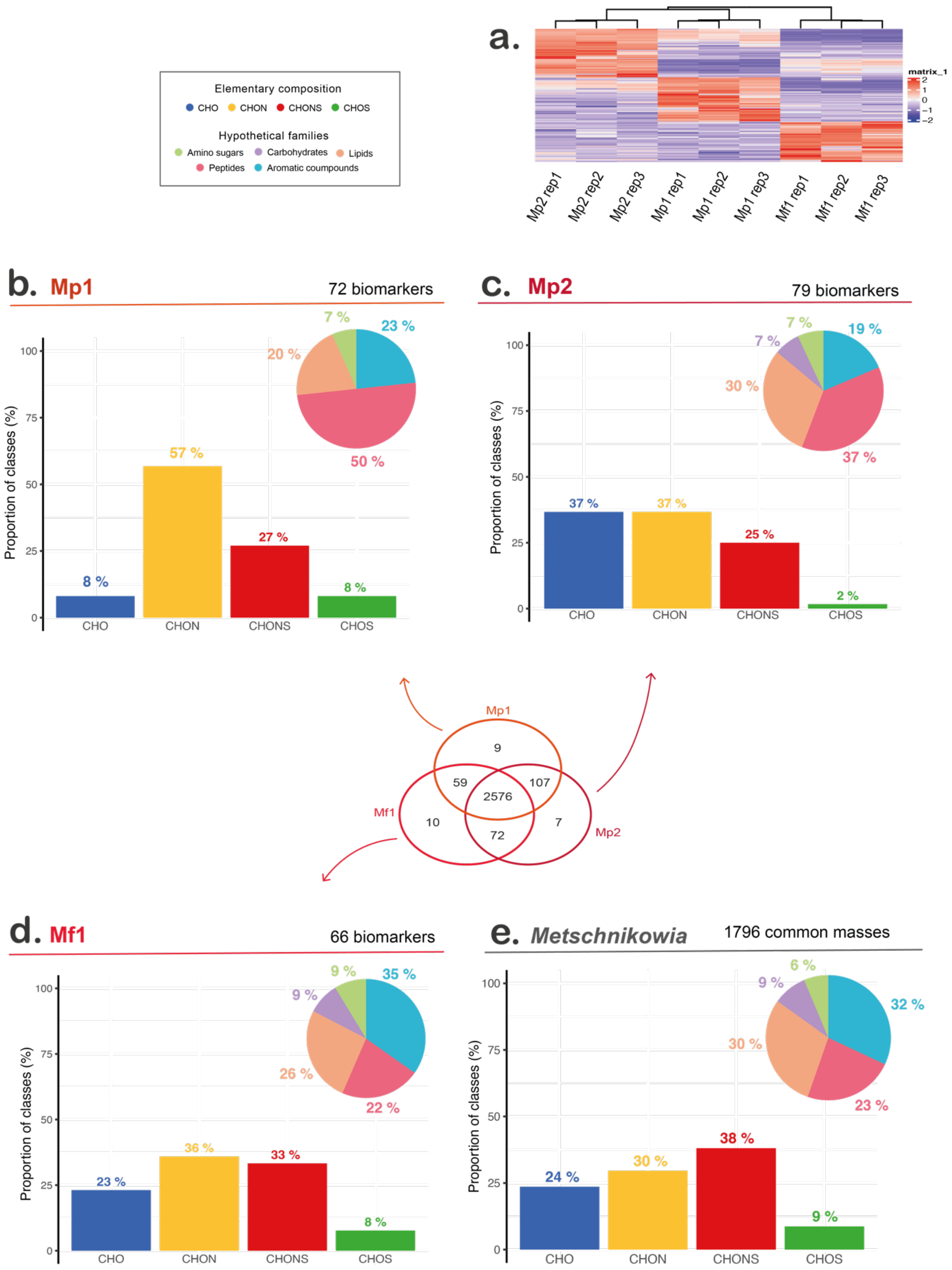

Figure S3| Comparative metabolomic analysis between the 3 *Metschnikowia* single cultures. (a) Heat map and ascendant hierarchical cluster of the three strains according to their biomarkers. Chemical composition of (a) Mp1 biomarker, (b) Mf1 biomarker, (c) Mp2 biomarker and (d) features common to the 3 *Metschnikowia* strains.

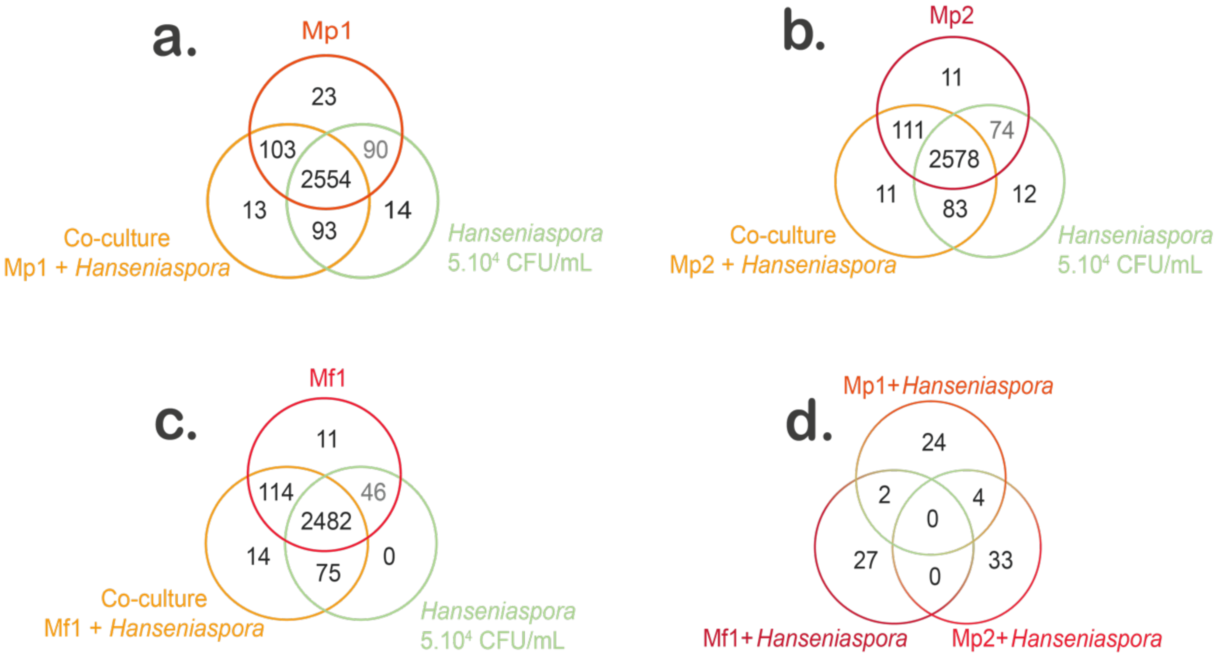

Figure S4 l Venn diagram for comparison of *Hanseniaspora* 5.10⁴ CFU/mL single culture and single culture of *Metschnikowia* and co-cultures of both species with (a) Mp1 strain, (b) Mp2 strain, (c) Mf1, and for (d) comparison of biomarkers of the three co-cultures.

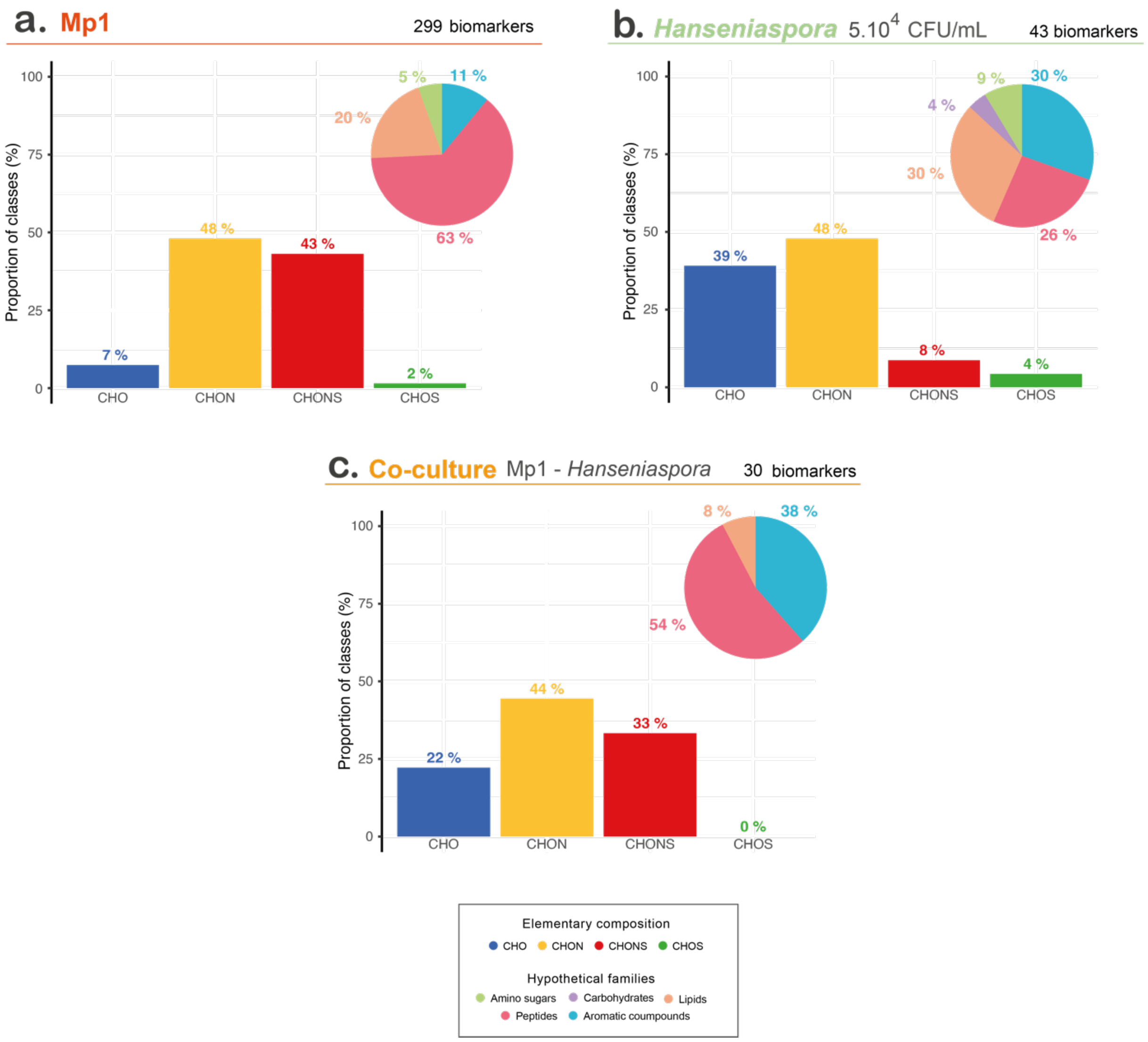

Figure S5 | Metabolomic analyses with hypothetical families, Van Krevelen diagram and elementary composition of (a) *M. pulcherrima* Mp1 strain in single culture, (b) *Hanseniaspora* mixture at an initial concentration of 5.10^4 CFU/mL, and (c) co-culture of Mp1 and *Hanseniaspora* mixture at an initial concentration of 5.10^4 CFU/mL.

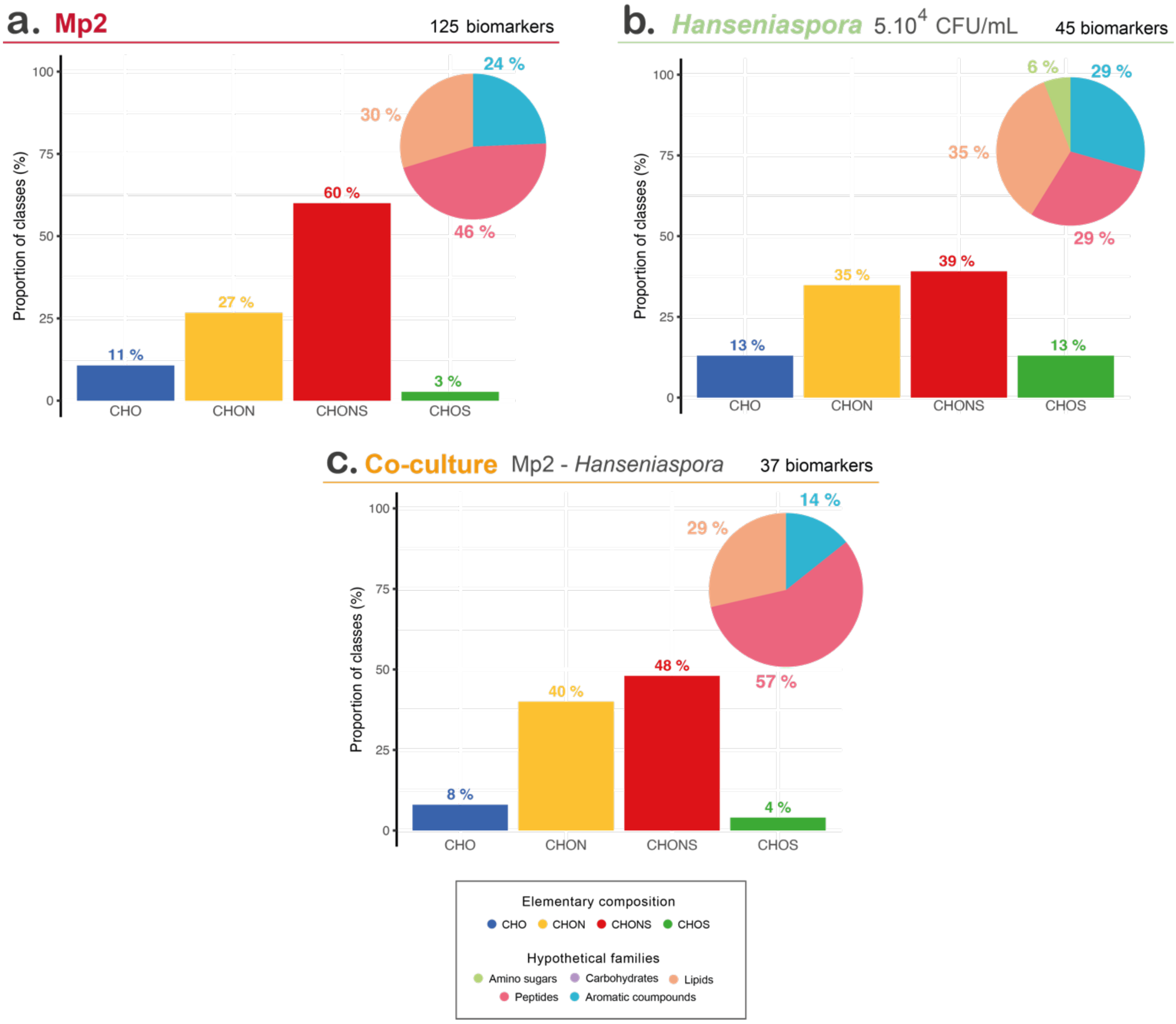

Figure S6 | Metabolomic analyses with hypothetical families, Van Krevelen diagram and elementary composition of (a) *M. pulcherrima* Mp2 strain in single culture, (b) *Hanseniaspora* mixture at an initial concentration of 5.10⁴ CFU/mL, and (c) co-culture of Mp2 and *Hanseniaspora* mixture at an initial concentration of 5.10⁴ CFU/mL.

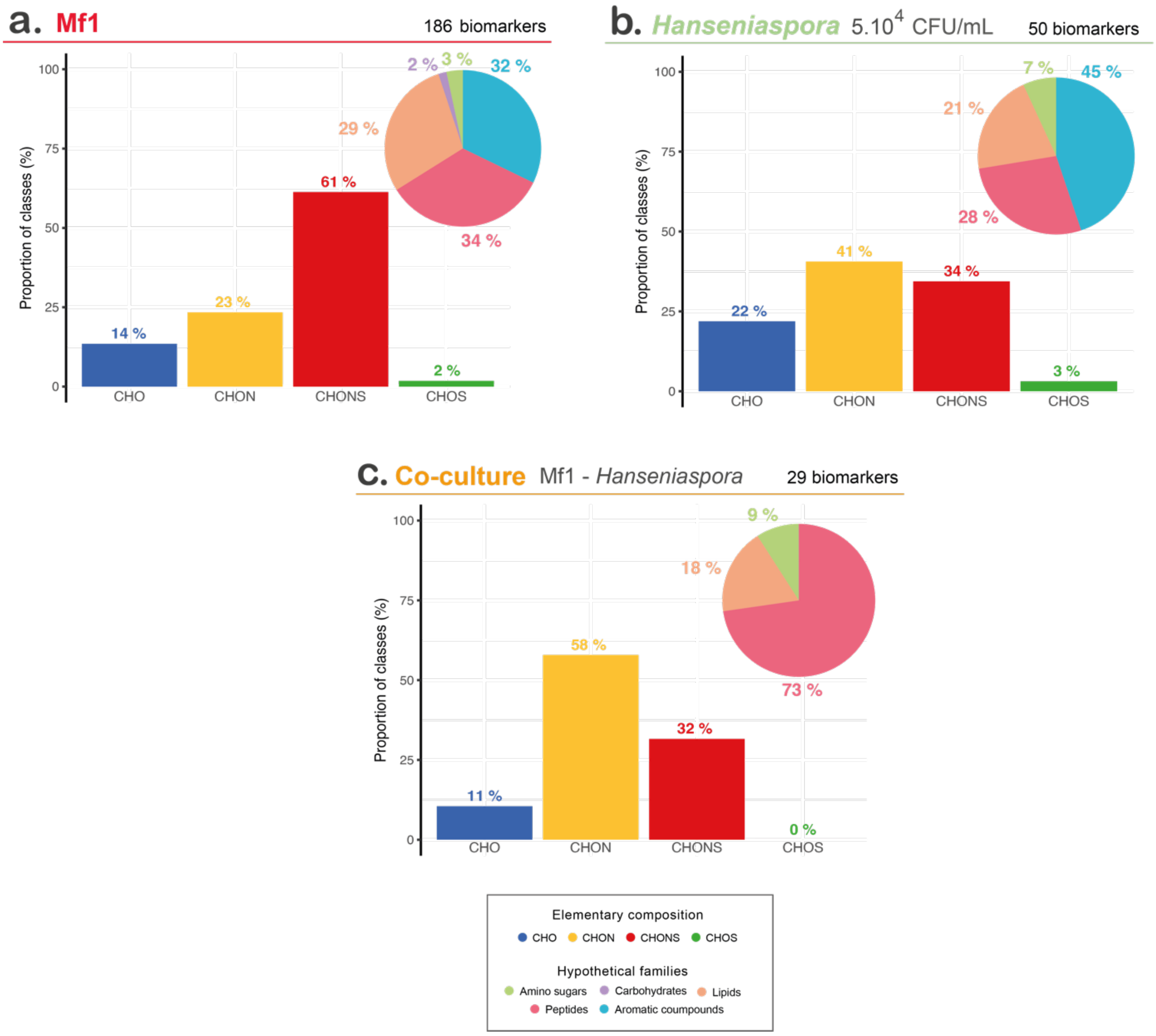

Figure S7: Metabolomic analyses with hypothetical families, Van Krevelen diagram and elementary composition of (a) *M. fructicola* Mf1 strain in single culture, (b) *Hanseniaspora* mixture at an initial concentration of 5.10^4 CFU/mL, and (c) co-culture of Mf1 and *Hanseniaspora* mixture at an initial concentration of 5.10^4 CFU/mL.
